# TRACC‐PHYSIO: Time‐Domain Resolution‐Aligned Cross‐Correlation to Estimate PHYSIOlogical Coupling and Time Delays in Dynamic MRI

**DOI:** 10.1002/mrm.70446

**Published:** 2026-05-24

**Authors:** Adam M. Wright, Jianing Zhang, Yunjie Tong, Qiuting Wen

**Affiliations:** ^1^ Department of Radiology and Imaging Sciences Indiana University School of Medicine Indianapolis Indiana USA; ^2^ Weldon School of Biomedical Engineering Purdue University West Lafayette Indiana USA

**Keywords:** cardiac and respiratory pulsations, coupling strength, dynamic diffusion‐weighted imaging (dynDWI), functional MRI (fMRI), neurofluid dynamics, pulse time delays

## Abstract

**Purpose:**

To validate a method to assess cardiac and respiratory brain pulsations in slowly sampled dynamic MR scans. Cardiac and respiratory pulsations are key drivers of neurofluid circulation. However, resolving their temporal dynamics requires fast imaging, which is not achievable in many dynamic MR acquisitions.

**Methods:**

We systematically validated TRACC‐PHYSIO, a Time‐domain Resolution‐Aligned Cross‐Correlation framework for estimating physiological coupling and time delays in dynamic MRI. This method uses simultaneously recorded cardiac and respiratory waveforms as external references and applies time‐shifted cross‐correlation to estimate physiological coupling strength and relative pulse delay in dynamic MRI data. Two metrics are derived: Peak Coupling Coefficient (Peak CorrCoeff), which quantifies coupling strength, and TimeDelay, which estimates the relative arrival time of the physiological impulse in the brain. TRACC‐PHYSIO was first evaluated with in vivo fMRI (TR = 363 ms) by comparing the Peak CorrCoeff with physiological bandpower derived from spectral analysis. Performance was further assessed using simulations of realistically modeled dynamic MR signals across repetition time (TR = 50–3000 ms) and acquisition durations (60–360 s).

**Results:**

In fMRI, TRACC‐derived cardiac and respiratory Peak CorrCoeff were strongly associated with their respective spectrum‐derived physiological bandpower (all tests Pearson *r* > 0.90). In simulations, both Peak CorrCoeff and TimeDelay were estimated with no mean bias and low temporal errors across TRs and acquisition durations.

**Conclusion:**

TRACC‐PHYSIO is a validated time‐domain framework for quantifying cardiac and respiratory coupling strength and estimating millisecond‐scale relative pulse delays in standard dynamic MR acquisitions.

## 1 Introduction

Physiological brain pulsations play a crucial role in driving neurofluid circulation and waste clearance [1, 2, 3, 4, 5, 6]. Cardiac and respiratory oscillations contribute to the motion of blood [7, 8], cerebrospinal fluid (CSF) [9, 10, 11], and interstitial fluid [1]. The extent to which physiologic pulsations are transmitted to specific brain compartments, quantified as physiological coupling, determines their mechanical influence on fluid motion, a process critical to maintaining brain homeostasis. However, quantifying physiological coupling and pulse propagation delays within the brain remains challenging. Resolving cardiac and respiratory frequency components requires imaging repetition times (TR; defined here as the time between successive MR volumes) below 300–400 ms to satisfy Nyquist sampling criteria [12, 13]. Additionally, characterizing pulse transmission timing and relative delays benefits from even higher temporal resolution (on the order of 100 ms), requiring ultrafast imaging [1].

High‐temporal‐resolution MRI approaches have been used to characterize physiological pulsations. Phase‐contrast MRI (PC‐MRI) has quantified cerebral arterial‐to‐venous cardiac pulse delays, providing insight into pulse‐wave timing, but it is limited in spatial coverage [14, 15]. Ultrafast fMRI (TR = 100 ms) enables whole‐brain assessment of physiological pulsations via spectral analysis and tracking pulse propagation; however, this approach requires sequences that are not widely available [1, 16]. More broadly, existing methods for resolving physiological timing and coupling rely on high temporal resolution, which is not feasible in many dynamic MRI acquisitions. As a result, the temporal resolutions required to directly resolve physiological coupling and pulse propagation are uncommon in standard dynamic MRI (e.g., fMRI or dynamic diffusion‐weighted imaging), leaving these dynamics largely inaccessible in most MR datasets.

To overcome the temporal limitations of dynamic MRI, physiological information can be resolved using the method Time‐domain Resolution‐Aligned Cross‐Correlation to estimate PHYSIOlogical coupling and time delays in dynamic MRI (TRACC‐PHYSIO). TRACC‐PHYSIO leverages simultaneously recorded cardiac and respiratory waveforms as external references and applies time‐shifted cross‐correlation to estimate physiological coupling strength and relative delay in dynamic MRI data, without requiring ultrafast imaging. The cross‐correlation function yields two metrics: the Peak Coupling Coefficient (Peak CorrCoeff), which quantifies the strength of the coupling, and TimeDelay, which measures the relative pulse arrival time within the brain. Quantifying physiological coupling and pulse timing enables characterization of how pulsations propagate across intracranial compartments and provides insight into their influence on fluid dynamics. Alterations in coupling strength and propagation delay may serve as early indicators of impaired neurofluid dynamics in aging and neurodegenerative conditions [14, 17]. Prior work suggests physiological pulsations follow distinct intracranial pathways [1], and alterations in these pathways have been reported in Alzheimer's disease [18]. However, these observations required ultrafast imaging (TR = 100 ms). TRACC‐PHYSIO provides an alternative approach for quantifying pulse timing in conventional, slower imaging datasets. Our group has applied this approach to characterize cardiac and respiratory coupling and pulse timing in CSF using dynDWI [19, 20, 21], and to assess time delays between large cerebral arteries and venous sinus using fMRI [22], demonstrating measurable age‐related alterations in pulse transmission.

Despite prior applications of TRACC‐PHYSIO, its performance has not been systematically evaluated. Here, we combine in vivo experiments and simulations to evaluate three aspects of the method: (1) the physiological interpretability of Peak CorrCoeff, (2) the accuracy and consistency of Peak CorrCoeff and millisecond‐scale TimeDelay estimations, and (3) the effects of TR, scan duration, and physiological composition on performance.

## 2 Methods

### 2.1 TRACC‐PHYSIO Method

TRACC‐PHYSIO performs a cross‐correlation between a dynamic MRI signal (typically sampled at TR > 800 ms) and a simultaneously recorded physiological signal with a much higher sampling rate (i.e., 2.5 ms per sample, 400 Hz). It measures two key metrics: a coupling Correlation Coefficient (CorrCoeff) that quantifies the strength of temporal coupling between the MR and physiological signals and a TimeDelay that reflects the relative arrival time of the physiological impulse in the MR signal compared to the physiological recording site (e.g., brain vs. finger or chest). TRACC‐PHYSIO requires single‐shot readout data to enable temporal alignment with the physiological signal. It further assumes that the physiological pulsations exhibit similar waveform morphology between the MR and the reference signals.

**FIGURE 1:**
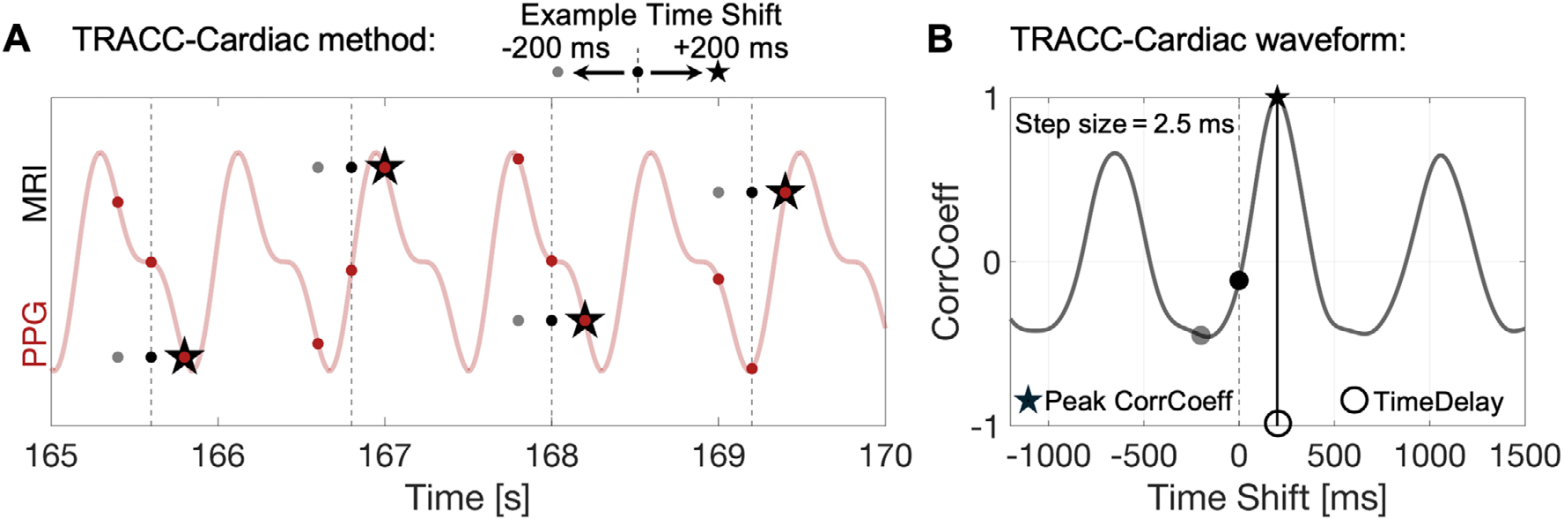
Overview of the TRACC‐PHYSIO method to measure peak cardiac coupling and pulse time delay. (A) An MR signal (black dots) and corresponding physiological signal (finger PPG, red waveform) at various temporal shifts. For visualization, the MR signal is shown translated along the time axis to depict example shifts of −200 ms (gray), 0 ms (black), and +200 ms (star). The red dots indicate the corresponding PPG samples used to compute the CorrCoeff at each shift. (B) The resulting cross‐correlation curve captures the degree of co‐fluctuation between the MR and PPG signals as a function of temporal shift. The three example shifts from panel A are marked on the curve to illustrate their corresponding correlation values (CorrCoeff). The Peak CorrCoeff and its associated temporal shift (TimeDelay) indicate when the strongest coupling occurs. In this example, a peak CorrCoeff of 1 indicates perfect coupling between the MR and PPG signals, with a TimeDelay of +200 ms, indicating that the MR signal leads the PPG signal by 200 ms. Note that the Peak CorrCoeff is higher than the neighboring side peaks in the TRACC‐Cardiac waveform; variability in heart rate leads to reduced correlation when aligning with pulses that are offset by one cycle. PPG, photoplethysmography.

In TRACC‐PHYSIO, temporal shifts (τ) are applied to the physiological waveform in increments equal to the physiological sampling interval (2.5 ms). For each shift, the Pearson correlation coefficient is computed between the MR signal and the shifted physiological signal evaluated at the MR acquisition times, without interpolating the MR time series (Figure 1A). Repeating this procedure across a range of shifts produces a cross‐correlation waveform–termed TRACC‐Cardiac or TRACC‐Respiratory waveforms–from which the peak correlation coefficient (Peak CorrCoeff) and corresponding TimeDelay are derived (Figure 1B). For visualization, Figure 1 shows MR samples sliding along the physiological waveform, which is mathematically equivalent to shifting the physiological signal (see the Appendix A for equations). The stepwise cross‐correlation and resulting TRACC‐Cardiac waveform construction are further summarized in Video S1.

### 2.2 Human in Vivo Implementation and Validation of TRACC‐PHYSIO

To validate that TRACC‐PHYSIO quantifies physiological contributions in MR signals, we applied the method to fast fMRI data (TR = 363 ms) that adequately sampled the cardiac and respiratory frequencies. TRACC‐derived Peak CorrCoeff values were compared with corresponding cardiac and respiratory spectral bandpower to assess physiological specificity and quantitative agreement.

#### 2.2.1 Participants

Data from 26 participants (mean age = 53.8 ± 12.8 years, range = 35–82 years) were used in this study. Data collection was approved by Indiana University's Institutional Review Board and written informed consent was obtained from all participants.

#### 2.2.2 Image Acquisition and Processing

fMRI was collected with a 3 T Siemens Prisma scanner and a 64‐channel head–neck coil. The fast fMRI was acquired with the following parameters: TR = 363 ms, echo time = 30 ms, flip angle = 35°, voxel size = 2.5 × 2.5 × 2.5 mm^3^, volumes = 500, MB factor = 8, with an acquisition time = 3 min and 2 s. Cardiac and respiratory signals were simultaneously recorded using a finger photoplethysmography (PPG) and a respiratory belt integrated with the scanner's physiological monitoring system.

The large cerebral arteries and the superior sagittal sinus (SSS) were directly segmented in fMRI space using a data‐driven automatic segmentation approach that leverages the pulsatile, reproducible cardiac‐induced signal fluctuations in large cerebral vessels [23].

#### 2.2.3 Voxel‐Wise Comparison of TRACC‐PHYSIO With Spectral Bandpower

Voxel‐wise TRACC‐PHYSIO was performed within large cerebral arteries, SSS, and gray matter (GM), yielding cardiac and respiratory Peak CorrCoeffs for each voxel. For comparison, voxel‐wise relative cardiac and respiratory bandpower were calculated from the power spectral density as a percentage of the total bandpower within 0–1.38 Hz (Nyquist Frequency for TR = 363 ms). Cardiac bandpower was defined as the power within ±0.15 Hz of the participant's cardiac frequency, and respiratory bandpower was defined as the power within ±0.05 Hz of the participant's respiratory frequency. The linear association between the |Peak CorrCoeff| and the corresponding relative bandpower (cardiac or respiratory) was assessed using Pearson's correlation.

### 2.3 Simulation‐Based Validation of TRACC‐PHYSIO

#### 2.3.1 Physiological Signal Generation

**FIGURE 2:**
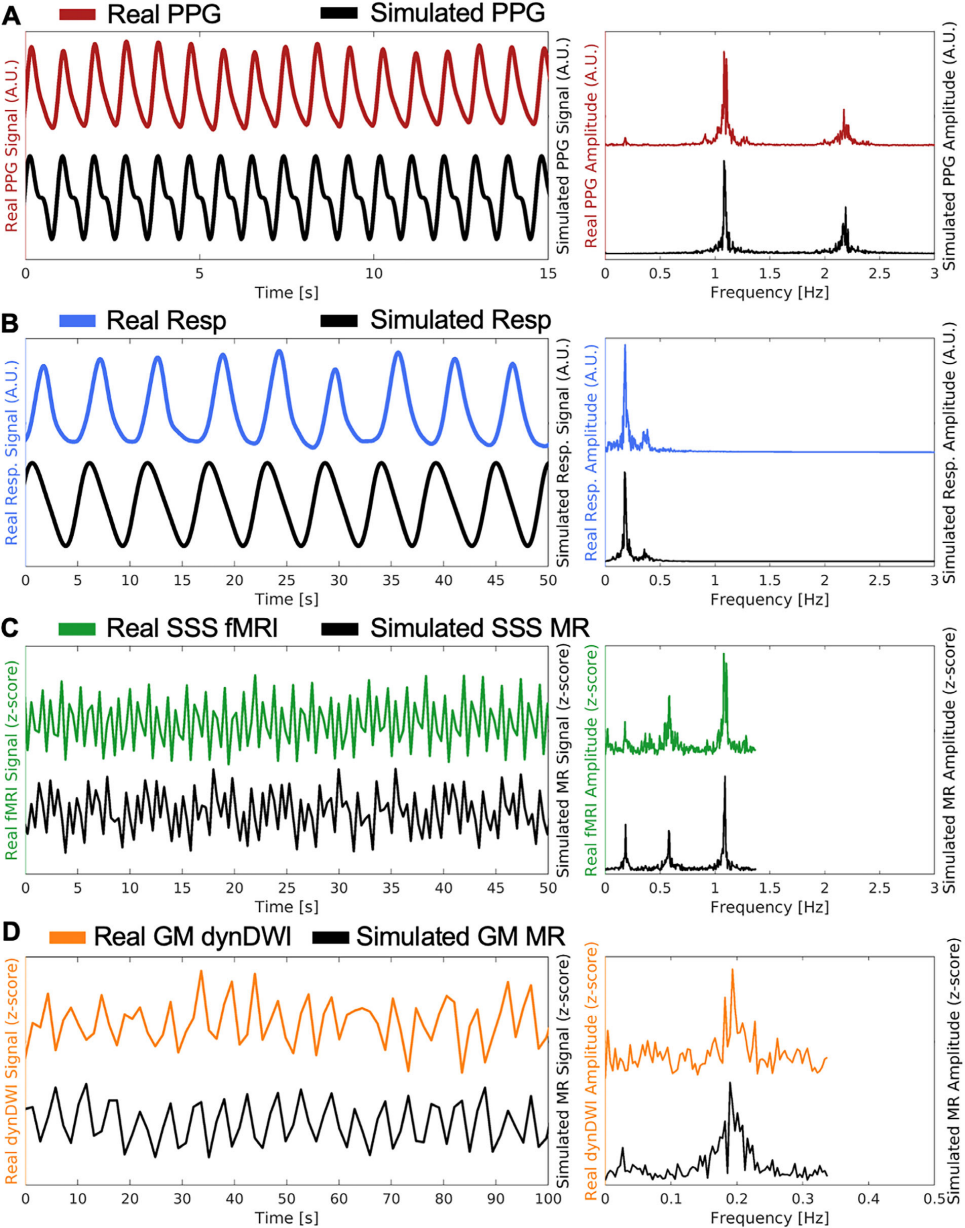
Representative real and simulated physiological and MR signals. (A) A representative real finger PPG signal (red) with a heart rate of 66 beats per minute and heart rate variability of 26 ms, and a simulated PPG with the same rate and variability (black), along with their amplitude spectrums. (B) A representative real respiration belt signal (blue) with a respiration rate of 12 breaths per minute and respiration rate variability of 420 ms, and a simulated respiratory signal with the same rate and variability (black), along with their amplitude spectrums. (C) A single voxel fast fMRI signal (TR = 363 ms) from the representative subjects' SSS and a simulated MR signal (black) generated with a cardiac‐to‐respiratory ratio of 2:1 (C:*R* = 2:1) with matched sampling rate and a temporal SNR of 20 dB. (D) A single voxel dynDWI signal (TR = 1.467 s) from the representative subjects' GM (GM) and a simulated MR signal (black) generated with a cardiac‐to‐respiratory ratio of 1:2 (C:*R* = 1:2) with matched sampling rate and a temporal SNR of 20 dB. dynDWI, dynamic diffusion‐weighted imaging; GM, gray matter; PPG, finger photoplethysmography; Resp, respiratory; SSS, superior sagittal sinus.

Synthetic finger PPG signals were generated by combining a smooth sine wave with a high‐frequency asymmetric component to best represent a cardiac beat with a sharp systolic upstroke, dicrotic notch, and gradual diastolic decay with a sampling frequency of 400 Hz. The heart rate (HR) and heart rate variability (HRV) were adjustable to represent any combination of physiologically plausible parameters in future simulations. Figure 2A illustrates a representative participant's finger PPG waveform with a HR of 65 beats per minute and a HRV of 26 ms (red) and a simulated finger PPG with the same HR and HRV (black).

Similarly, synthetic chest‐respiratory belt signals were generated using a sinusoidal waveform with an asymmetry to reflect the natural variability in inspiratory and expiratory durations (more time spent in expiration) with a sampling frequency of 400 Hz. The respiratory rate (RR) and respiratory rate variability (RRV) were adjustable to reflect any physiologically plausible breathing patterns. Figure 2B illustrates a representative participant's respiratory belt waveform with an RR of 12 breaths per minute and RRV of 420 ms (blue) and a simulated respiratory belt waveform with the same RR and RRV (black).

#### 2.3.2 Synthetic MR Signal Generation

To generate synthetic MR signals with physiologically driven dynamics, we summed simulated cardiac and respiratory waveforms using specified cardiac‐to‐respiratory ratios (C:R) and added temporal noise (tSNR = 20 dB). All simulated MR signals were initially generated at the physiological signal sampling rate of 400 Hz and an acquisition time of 600 s, then downsampled and truncated to emulate different TRs and acquisition times. Two representative examples are shown alongside real data (Figure 2C,D). In Figure 2C, a representative SSS voxel from a fast fMRI scan (TR = 363 ms) is shown with a simulated MR signal constructed with C:*R* = 2:1. In Figure 2D, a representative GM voxel from a low b‐value (150 s/mm^2^) dynDWI acquisition (T*R* = 1.467 s) is shown with a simulated MR signal with C:R = 1:2. These examples illustrate that the simulated MR signals can closely mirror relevant real MR signals with varying physiological dominance and imaging speeds.

#### 2.3.3 Simulation Experiments

In a simplified simulation, a noise‐free MR signal with varying TRs (TR = 0.05, 0.8, 1.6, and 2.4 s) and an acquisition time of 300 s was simulated to contain equal‐amplitude cardiac and respiratory components (C:*R* = 1:1). The MR cardiac component was time‐shifted to lead the cardiac physiological signal by 200 ms. Then, TRACC‐Cardiac was applied to estimate the Peak CorrCoeff and TimeDelay between the two signals. This simulation provides a concise evaluation of how TRACC‐Cardiac performs across different TRs.

In‐depth simulations were conducted to evaluate TRACC‐PHYSIO's performance under complex, real‐world conditions, incorporating the following factors: (1) *MR signal noise*: Random noise was added to the MR signals to achieve a temporal signal‐to‐noise ratio (tSNR) of 20 dB. A tSNR of 20 dB was selected as a conservative estimate based on published tSNR values in conventional fMRI acquisitions [24]. Empirical comparisons between our simulated signals and the in vivo fMRI/dynDWI datasets (Figure 2C,D) indicated that the selected noise level yields signal features in both the time and frequency domains comparable to the real data (Figure 2C,D). (2) *Cardiac‐to‐respiratory composition*: MR signals were simulated with three different cardiac‐to‐respiratory ratios, C:*R* = 2:1, 1:1, and 1:2. These ratios represent physiologically relevant regimens, including cardiac dominance, equal cardiac‐respiratory contribution, and respiratory dominance. Cardiac dominance is typical in large vascular regions [25], whereas respiratory dominance has been observed in parenchymal fluid motion [21]. For completeness, sensitivity analyses over a wider C:R range (4:1–1:4) are provided in the supplemental material (Figures S5 and S6). (3) *TR and acquisition time*: Simulations spanned TR values from 0.05 to 3 s (in 0.025 s increments) and acquisition times from 60 to 580 s (in 20 s increments) to assess their impact on TRACC performance. (4) *Physiological variability*: Cardiac and respiratory waveforms were generated with varying HR, HRV, RR, and RRV, based on predefined ranges that represent common physiological rates (Table 1). (5) *Variations in physiological time shift*: Randomized time shifts between MR and physiological signals were introduced to simulate natural biological variability in TimeDelay, with ranges informed by prior studies [19, 20, 21, 22].

**TABLE 1:** Physiological parameter ranges used in simulations.

Physiological parameter	Simulation ranges
Heart rate (HR) [bpm]	60–80
Heart rate variability (HRV) [ms]	25–60
Applied cardiac TimeDelay [ms]	150–250
Respiration rate (RR) [bpm]	12–16
Respiration rate variability (RRV) [ms]	400–600
Applied respiratory TimeDelay [ms]	−700 to −500

*Note:* Heart rate variability (HRV) and respiration rate variability (RRV) were defined as the standard deviation of beat‐to‐beat intervals and breath‐to‐breath intervals, respectively.

Abbreviation: Bpm, beats per minute or breaths per minute.

For each MR signal configuration (defined by a particular C:R ratio, TR, and acquisition time), 5000 permutations were performed. In each permutation, randomized HR, HRV, RR, and RRV were used to generate the physiological signals to mimic an individual's variability, the ground‐truth TimeDelay was randomized, and random noise was added to the MR signal. TRACC‐PHYSIO was then applied to estimate the Peak CorrCoeff and TimeDelay between the MR signal and each physiological signal (TRACC‐Cardiac and TRACC‐Respiratory). TimeDelay error was computed as the difference between the ground‐truth and the TRACC‐estimated TimeDelay.

## 3 Results

### 3.1 Human fMRI Results: Peak CorrCoeff Tracks Physiological Signal Power

**FIGURE 3:**
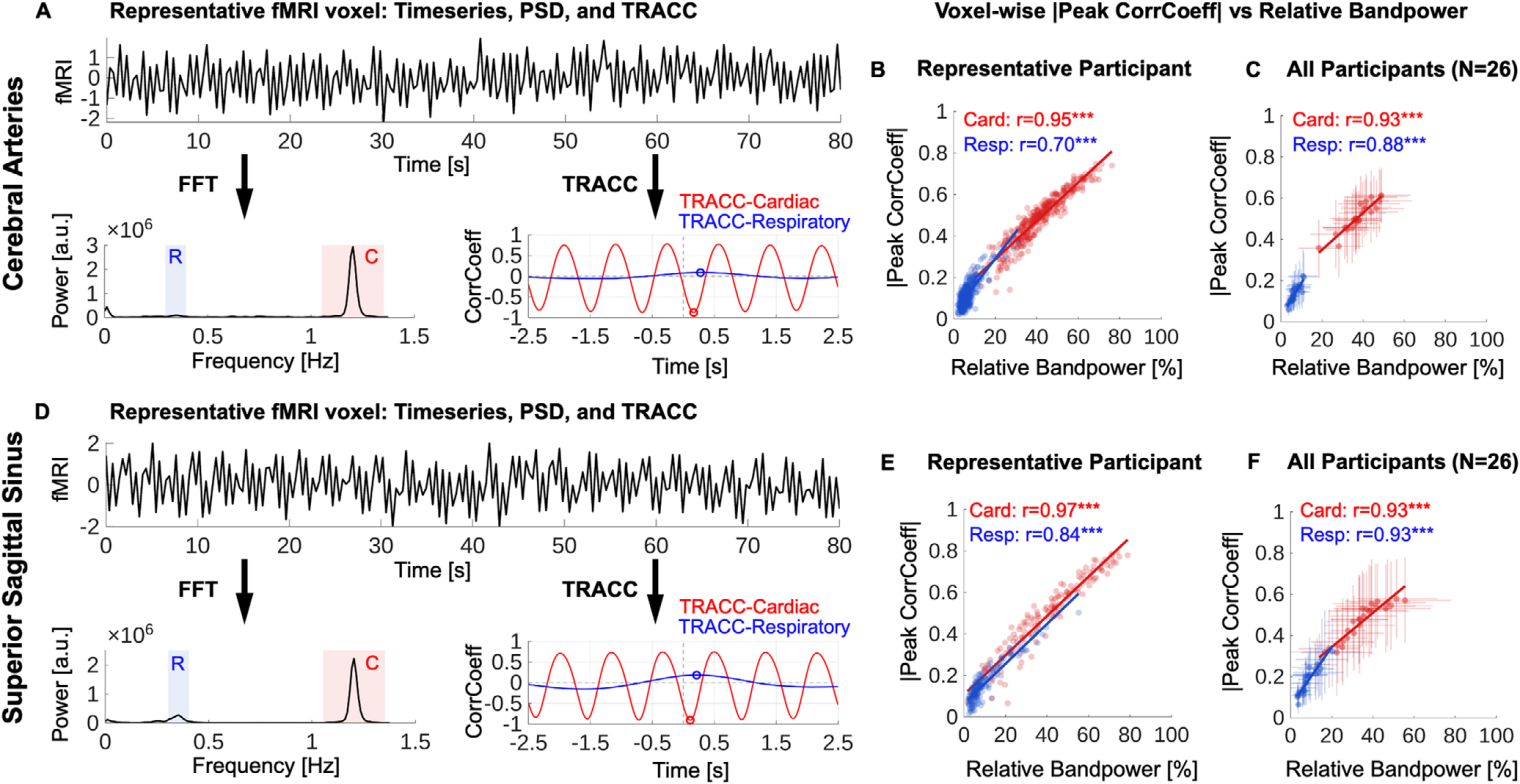
TRACC‐PHYSIO Peak CorrCoeffs scaled linearly with spectrum‐drived relative physiological power in fast fMRI vascular signals. (A) Representative single‐voxel fMRI timeseries from a cerebral artery (top), its power spectrum (left), and TRACC‐PHYSIO waveforms (right). (B) The Peak CorrCoeff versus relative bandpower for cardiac (red) and respiratory (blue) components across all cerebral artery voxels in a representative participant. (C) The mean Peak CorrCoeff vs. relative bandpower for cardiac (red) and respiratory (blue) components for the cerebral arteries in all participants. (D) Representative single‐voxel fMRI timeseries from the SSS (top), its power spectrum (left), and TRACC‐PHYSIO waveforms (right). (E) The Peak CorrCoeff versus relative bandpower for cardiac (red) and respiratory (blue) components across all SSS voxels in a representative participant. (F) The mean Peak CorrCoeff versus relative bandpower for cardiac (red) and respiratory (blue) components for the SSS in all participants. Card & C, cardiac; Resp & R, respiratory; ROI, region of interest; SSS, superior sagittal sinus. ***p < 0.001.

Absolute Peak CorrCoeff (|Peak CorrCoeff|) was positively associated with relative physiological bandpower in both the cerebral arteries and the SSS (Figure 3). Representative single‐voxel arterial and SSS fMRI time series, their power spectra, and TRACC‐PHYSIO results demonstrate that high cardiac bandpower corresponded to |Peak CorrCoeff| approaching 1 (Artery—Figure 3A; SSS—Figure 3D). Across all arterial and SSS voxels within a representative participant, linear relationships were observed between |Peak CorrCoeff| and relative physiological bandpower (Artery—Figure 3B: Card *r* = 0.95, *p* < 0.001; Resp *r* = 0.70, *p* < 0.001; SSS—Figure 3E: Card *r* = 0.97, *p* < 0.001; Resp *r* = 0.84, *p* < 0.001). At the group level, participants' mean |Peak CorrCoeff| values were likewise strongly correlated with mean relative physiological bandpower (Artery—Figure 3C: Card *r* = 0.93, *p* < 0.001; Resp *r* = 0.88, *p* < 0.001; SSS—Figure 3F: Card *r* = 0.93, *p* < 0.001; Resp *r* = 0.93, *p* < 0.001). In GM, where low‐frequency oscillations dominate, the TRACC CorrCoeff values for cardiac and respiratory components were lower than within the vascular regions, as expected. Peak CorrCoeff remained positively associated with relative physiological bandpower, indicating that TRACC‐PHYSIO remains sensitive to physiological signal contributions even in the presence of additional signal components (Figure S1: Group‐level, Card *r* = 0.58, *p* < 0.01, Resp *r* = 0.94, *p* < 0.001).

In addition to the Peak CorrCoeff, TRACC‐PHYSIO estimates TimeDelay, for which no in vivo ground truth exists. The accuracy of the TimeDelay estimation was evaluated using simulations, as reported below.

### 3.2 TRACC‐PHYSIO Performance Across Variable TRs in Simple Simulations

**FIGURE 4:**
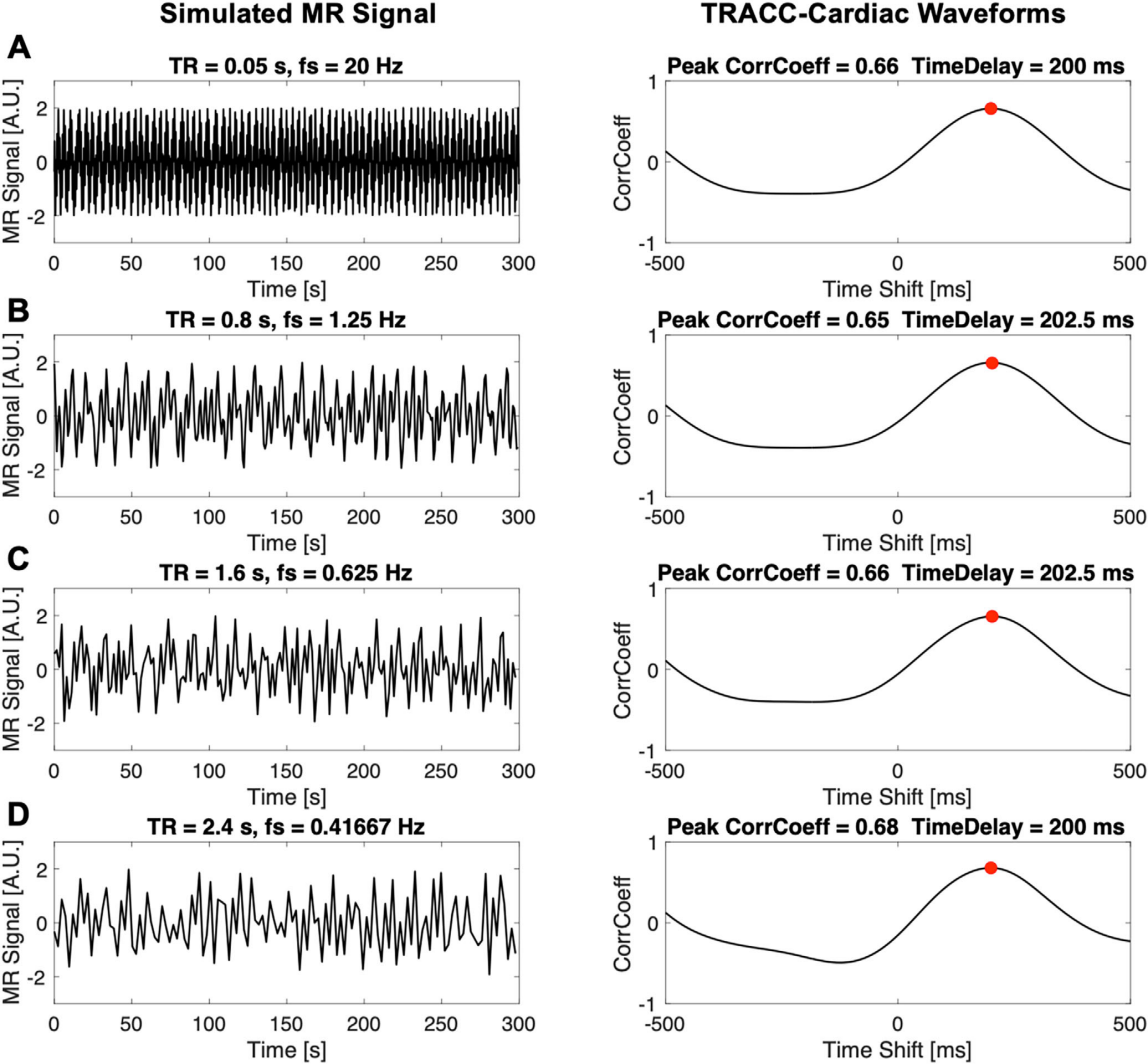
TRACC‐PHYSIO demonstrated high performance across variable TRs in simplified simulations. TRACC‐Cardiac was applied to simulated MR signals with equal cardiac and respiratory components with a ground‐truth cardiac TimeDelay of 200 ms. Peak CorrCoeff and TimeDelay estimates were consistent across short (A: TR = 0.05 s) and longer TRs (B: TR = 0.8 s; C: TR = 1.6 s; D: TR = 2.4 s).

In the noise‐free, simplified simulations with equal amplitude physiological components (C:*R* = 1:1), TRACC‐Cardiac produced consistent results across TRs ranging from 0.05 to 2.4 s (Figure 4). At TR = 0.05 s, it identified a Peak CorrCoeff of 0.66 and a TimeDelay of 200 ms (Figure 4A). With longer TRs of 0.8, 1.6, and 2.4 s, TRACC‐Cardiac yielded Peak CorrCoeffs between 0.65 and 0.68 and TimeDelays between 200 and 202.5 ms (Figure 4B–D), differing no more than 0.02 in Peak CorrCoeff and 2.5 ms in TimeDelay from the TR = 0.05 s baseline. This simulation demonstrates that TRACC‐Cardiac's accuracy and robustness at long TRs.

### 3.3 Robustness to Varying Physiological Components in Simulations

**FIGURE 5:**
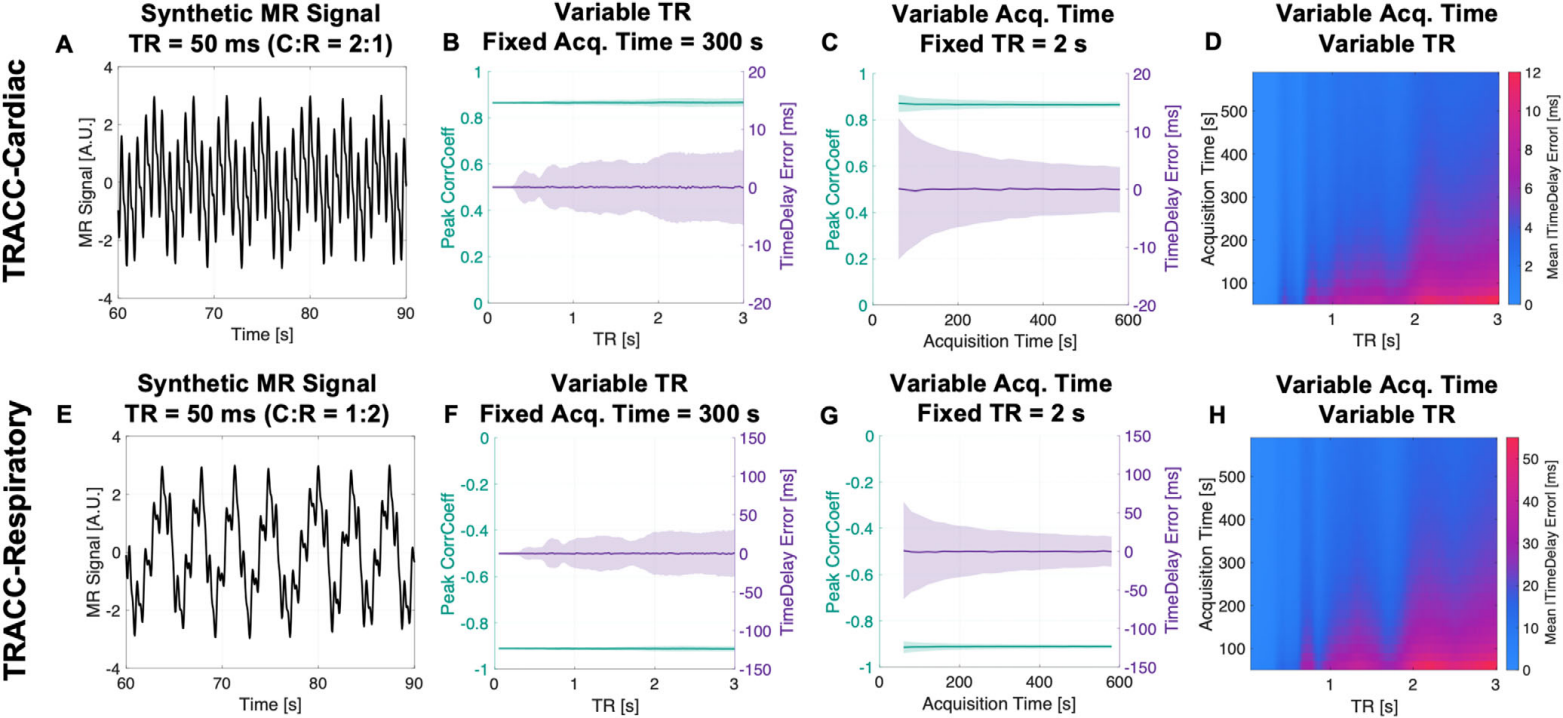
TRACC‐PHYSIO demonstrated robust performance across varying TRs and acquisition times when the target physiological component was dominant in the MR signal. Top row (A–D): TRACC‐Cardiac results for MR signals with C:*R* = 2:1. Bottom row (E–H): TRACC‐Respiratory results for MR signals with C:*R* = 1:2. From left to right: (A and E) Example synthetic MR signals with TR = 50 ms. (B and F) The mean (solid line) and standard deviation (shaded) of the Peak CorrCoeff and TimeDelay error with increasing TRs and a fixed acquisition time of 300 s. (C and G) The mean (solid line) and standard deviation (shaded) of the Peak CorrCoeff and TimeDelay error with increasing acquisition time and a fixed TR of 2 s. (D and H) Heatmap of the mean absolute TimeDelay error across all combinations of TRs and acquisition times. 5000 permutations were completed for each combination of TR and acquisition time. C:R, cardiac‐to‐respiratory ratios.

In simulations where the physiological coupling being solved for dominates the MR signal (i.e., C:*R* = 2:1 for TRACC‐Cardiac and C:*R* = 1:2 for TRACC‐Respiratory), TRACC‐PHYSIO yielded high Peak CorrCoeffs (> |0.8|) and accurately measured TimeDelays across various TRs and acquisition times (Figure 5). Across all simulations, both Peak CorrCoeff and TimeDelay measures had no mean error bias.

In both TRACC‐Cardiac and TRACC‐Respiratory, across all TRs with a fixed acquisition time of 300 s, the mean Peak CorrCoeff and mean TimeDelay error remained consistent, with values of 0.87 and 0 ms for TRACC‐Cardiac and −0.91 and 0 ms for TRACC‐Respiratory (Figure 5B,F). In TRACC‐Cardiac, the standard deviation of the TimeDelay error increased starting at TR = 0.275 s, just before cardiac aliasing would occur in the frequency domain; however, the errors never exceeded ±6.5 ms. In TRACC‐Respiratory, standard deviation increased at TR = 0.3 s and again at TR = 0.6 s, but the errors never exceeded ±30 ms. With a fixed TR = 2 s and increasing acquisition time, the mean Peak CorrCoeff and mean TimeDelay error remained consistent in both TRACC‐Cardiac and TRACC‐Respiratory, and the measures' standard deviation decreased as the acquisition time increased (Figure 5C,G). The mean absolute TimeDelay errors for TRACC‐Cardiac and TRACC‐Respiratory across all combinations of TR and acquisition time are summarized in Figure 5D,H, demonstrating that TimeDelay errors remained low across most acquisition parameters and were higher in simulation with longer TR and shorter scan durations.

**FIGURE 6:**
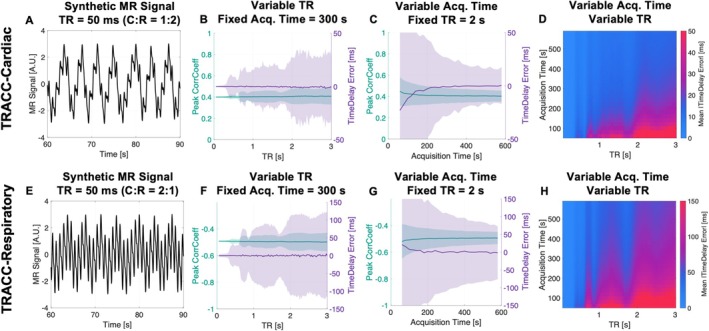
TRACC‐PHYSIO maintained reasonable accuracy even when the target physiological component was nondominant in the MR signal. Top row (A–D): TRACC‐Cardiac results for MR signals with C:*R* = 1:2. Bottom row (E‐H): TRACC‐Respiratory results for MR signals with C:*R* = 2:1. From left to right: (A and E) Example synthetic MR signals with TR = 50 ms. (B and F) The mean (solid line) and standard deviation (shaded) of the Peak CorrCoeff and TimeDelay error with increasing TRs and a fixed acquisition time of 300 s. (C and G) The mean (solid line) and standard deviation (shaded) of the Peak CorrCoeff and TimeDelay error with increasing acquisition time and a fixed TR of 2 s. (D and H) Heatmap of the mean absolute TimeDelay error across all combinations of TRs and acquisition times. 5000 permutations were completed for each combination of TR and acquisition time. C:R, cardiac‐to‐respiratory ratios.

In simulations where the physiological coupling being solved for was nondominant in the MR signal (i.e., C:*R* = 1:2 for TRACC‐Cardiac and 2:1 for TRACC‐Respiratory), TRACC‐PHYSIO yielded moderate Peak CorrCoeffs (∼|0.4−0.5|) and maintained accurate TimeDelay estimates across various TRs and acquisition times (Figure 6). As expected, uncertainty in both Peak CorrCoeff and TimeDelay was higher compared to simulations with dominant coupling (Figure 6B vs. Figures 5B, 6F vs. Figure 5F). Slight biases in TimeDelays emerged at short acquisition times (< 200 s) with a TR of 2 s (Figure 6C), but these biases disappeared with longer acquisition times. Heatmaps of mean absolute TimeDelay errors across TR and acquisition time combinations (Figure 6D,H) demonstrate that the most accurate performance was achieved with shorter TRs and longer acquisition times.

In simulations where the physiological components had equal amplitude in the MR signal (i.e., C:*R* = 1:1), TRACC‐PHYSIO yielded Peak CorrCoeffs in the range of |0.6−0.8| and accurately measured TimeDelays across various TRs and acquisition times, which are fully summarized in Figure S2. Across all simulations, the errors observed in TRACC‐PHYSIO were between those observed in solving for the dominant and nondominant physiological components.

### 3.4 Performance in Simulated 5 Min Scans

The simulation results in a typical acquisition time of 300 s at representative TRs and varying physiological signal components are summarized in Table 2. In both TRACC‐Cardiac and TRACC‐Respiratory, absolute Peak CorrCoeff values progressively decreased as the relative contribution of the target physiological signal decreased. Using TRACC‐Cardiac in cardiac‐dominant signals with a long TR of 3 s, the mean absolute TimeDelay error was 5.1 ms. Errors were slightly higher when using TRACC‐Respiratory in respiratory‐dominant signals with a long TR of 3 s, yielding a mean absolute TimeDelay error of 24.0 ms. When solving for nondominant physiological components, the results yielded higher TimeDelay errors: 20.4 ms for TRACC‐Cardiac and 92.8 ms for TRACC‐Respiratory. In all scenarios, TimeDelay errors progressively decreased with shorter TRs.

**TABLE 2:** Summary of TRACC‐PHYSIO performance in 5 min scans.

TRACC‐cardiac		TRACC‐respiratory	
Cardiac‐to‐respiratory ratio (C:R)	TR (s)	Peak CorrCoeff mean (5%–95%)	Absolute TimeDelay error (ms) mean (5%–95%)	Peak CorrCoeff mean (5%–95%)	Absolute TimeDelay error (ms) mean (5%–95%)
2:1	0.05	0.87 (0.86, 0.87)	0.0 (0, 0)	−0.49 (−0.49, −0.49)	2.6 (0, 7.5)
	0.8	0.87 (0.85, 0.88)	2.6 (0, 7.5)	−0.49 (−0.56, −0.43)	45.0 (2.5, 141.3)
	1	0.87 (0.85, 0.88)	2.8 (0, 7.5)	−0.49 (−0.56, −0.42)	52.0 (2.5, 153.8)
	2	0.87 (0.84, 0.89)	4.0 (0, 10.0)	−0.50 (−0.60, −0.40)	80.9 (5.0, 230.0)
	3	0.87 (0.84, 0.89)	4.9 (0, 12.5)	−0.50 (−0.61, −0.38)	92.3 (7.5, 245.0)
1:1	0.05	0.66 (0.66, 0.66)	0.0 (0, 0)	−0.75 (−0.75, −0.75)	1.4 (0, 2.5)
	0.8	0.66 (0.61, 0.70)	5.4 (0, 15.0)	−0.75 (−0.78, −0.72)	21.7 (2.5, 67.5)
	1	0.66 (0.61, 0.71)	5.6 (0, 15.0)	−0.75 (−0.78, −0.71)	27.2 (2.5, 80.0)
	2	0.66 (0.58, 0.73)	8.1 (0, 22.5)	−0.75 (−0.80, −0.69)	38.8 (2.5, 112.5)
	3	0.66 (0.58, 0.73)	9.7 (0, 25.0)	−0.75 (−0.80, −0.69)	46.6 (2.5, 120.0)
1:2	0.05	0.40 (0.40, 0.40)	0.0 (0, 0)	−0.91 (−0.91, −0.91)	0.7 (0, 2.5)
	0.8	0.40 (0.33, 0.47)	11.3 (0, 32.5)	−0.91 (−0.92, −0.90)	11.6 (0, 37.5)
	1	0.40 (0.32, 0.48)	11.5 (0, 32.5)	−0.91 (−0.92, −0.90)	13.4 (0, 40.0)
	2	0.41 (0.28, 0.52)	17.2 (0, 45.0)	−0.91 (−0.93, −0.89)	20.6 (2.5, 57.5)
	3	0.41 (0.28, 0.53)	20.8 (2.5, 55.0)	−0.91 (−0.93, −0.89)	23.6 (2.5, 60.0)

*Note:* Each row summarizes simulation results of 5000 permutations for a specific cardiac‐to‐respiratory (C:R) physiological composition and TR. For each permutation, the applied TimeDelay was randomly selected between 150 and 250 ms (mean = 200 ms) for cardiac and between −700 and −500 ms for respiration (mean = −600 ms), with the heart rate, heart rate variability, respiratory rate, and respiratory rate variability randomized within a physiologically plausible range. Gaussian noise was added to achieve a temporal signal‐to‐noise ratio of 20 dB.

## 4 Discussion

Through real MR data and simulations, we validated that TRACC‐PHYSIO can effectively assess physiological coupling and pulsation arrival timing in dynamic MR. First, we demonstrated that the Peak CorrCoeff in vascular fMRI was closely associated with relative physiological bandpower. Next, we comprehensively characterized the performance of TRACC‐PHYSIO in MR signals with mixed physiological components. Below, we interpret the Peak CorrCoeff and TimeDelay metrics and discuss how MR acquisition parameters and the physiological composition influence TRACC‐PHYSIO's performance.

Using fast fMRI data to resolve both cardiac and respiratory components, we showed that TRACC‐derived CorrCoeff values scaled with their corresponding relative physiological bandpower. Notably, this relationship persisted even in the presence of other signal components, such as dominant low‐frequency oscillations in GM. This finding supports the interpretation that TRACC‐PHYSIO provides an effective surrogate measure for quantifying cardiac and respiratory contributions to dynamic MR signal variation. These results are also consistent with the theoretical basis of the method, in which CorrCoeff quantifies the degree to which the MRI signal dynamics co‐fluctuate with physiological reference signals. Its square (CorrCoeff^2^ or *r* ^2^) reflects the proportion of MR signal variance explained by the physiological input. A higher CorrCoeff indicates stronger co‐fluctuation and provides a valuable metric for assessing physiological contributions to MR signals, particularly in long TR acquisitions. In neurofluid imaging modalities such as fMRI and dynDWI, long TRs lead to aliasing of cardiac (∼TR > 400 ms) and respiratory (∼TR > 1800 ms) frequencies, thereby hindering spectral analysis. Consequently, the physiological drivers across different brain regions are still under debate [26]. Our simulation results demonstrate that TRACC‐Cardiac and TRACC‐Respiratory can be applied to these MR signals, with Peak CorrCoeff offering a robust measure of cardiac and respiratory contributions. Recent application of TRACC‐PHSYIO suggests that parenchymal fluid dynamics are strongly coupled to respiration, whereas the fluid motion within the subarachnoid space is more closely linked to cardiac pulsatility [21].

The TimeDelay reflects the latency between the physiological pulsation arrival in the brain and the peripheral physiological signal. A key advantage of the TRACC‐derived TimeDelay is its high millisecond‐scale temporal resolution, which matches the sampling rate of the physiological signal recordings. This fine resolution enables the detection and quantification of fast‐traveling physiological waves to and within the brain. For example, TimeDelay estimates have revealed a 244 ms delay in cardiac‐driven cerebrospinal fluid pulsations in the brain and the corresponding finger blood pulse [20], an 84 ms cardiac transmission from the subarachnoid space to the cortical surface [19], and a ∼80 ms cerebral arterial–venous cardiac pulse delay [22]. The arterial–venous delay measured using the TRACC‐PHYSIO TimeDelay was consistent with values reported with PC‐MRI, [15, 27] supporting the TimeDelay's physiological validity. Further validation of TimeDelay‐derived measures should be performed as new techniques are developed.

The high temporal precision also allows for assessment of pulse propagation. Another advantage of the TRACC‐derived TimeDelay is that it is measured in the time domain, preserving the true timing relationships between signals. In contrast, phase delays measured with retrospective rebinning approaches can introduce phase distortions by forcing cycles of variable length (from natural physiological variation) into a fixed phase scale, which may cause temporal offset errors. However, TRACC‐PHYSIO requires a different consideration: to avoid misalignment with neighboring pulses under noisy conditions, the TimeDelay search window should be restricted to one‐half of a physiological period and empirically centered on a physiologically plausible time based on group averages. Given the advantages and specific considerations of TRACC‐PHYSIO, it holds great promise for broad applications to advance our understanding of neurofluid pulse timing, with potential applications in studies of aging and disease.

Our simulations indicate that both TR and acquisition time affect the accuracy of Peak CorrCoeff and TimeDelay measures. As expected, short TR and longer acquisition time improve accuracy. Overall, Peak CorrCoeff was very stable across all MR parameters (Figures S3 and S4). TimeDelays errors in both TRACC‐Cardiac and TRACC‐Respiratory increased around a TR of 300 ms, coinciding with the onset of cardiac aliasing. Under typical MR acquisition paradigms (TRs of 1–3 s; 5‐min acquisition), TimeDelays remained reasonably accurate. Specifically, when TRACC‐Cardiac was applied to a C:*R* = 2:1 signal, the mean absolute TimeDelay errors were 2.9 and 4.0 ms at TRs of 1 and 2 s, respectively. For TRACC‐Respiratory, with a C:*R* = 1:2 signal, the corresponding errors were 13.8 and 20.2 ms. The TimeDelay errors were independent of the ground‐truth TimeDelay (results not shown). For representative cardiac and respiratory TimeDelays of 200 and −600 ms, relative errors ranged from 1.5% to 2% for TRACC‐Cardiac and 2.3%–3.4% for TRACC‐Respiratory, across TRs of 1–2 s. The higher error in TRACC‐Respiratory likely stems from the lower respiratory frequency: over the same scan duration, respiratory cycles are 4–6 fold fewer than cardiac cycles, yielding less precise signal alignment. Based on these simulations, TRACC‐PHYSIO is expected to perform reliably with TRs ≤ 2 s and acquisition times ≥ 180 s. For longer TRs (> 2 s), performance can be maintained with extended acquisition times (≥ 300 s), although uncertainty increases as physiological signal contributions decrease.

Additionally, the physiological composition influences the accuracy of TRACC‐PHYSIO results, particularly in TimeDelay estimates. When solving for the nondominant physiological components, relative TimeDelay errors increased when compared to solving for the dominant physiological component. This suggests that Peak CorrCoeff, which reflects the level of physiological dominance, can serve as an indicator of confidence in the TimeDelay measurement. Supporting this, a supplementary regression analysis showed a significant association between TimeDelay error and Peak CorrCoeff (see Tables S1 and S2). In summary, CorrCoeff not only reflects physiological coupling strength (see Figures S5 and S6) but also provides a measure of certainty of the TimeDelay measurement. Despite greater variability in TimeDelay for nondominant components, TRACC‐PHYSIO introduced no systematic bias in either Peak CorrCoeff or TimeDelay estimates. The symmetric error distribution ensures valid and interpretable group‐level results, with estimates that converge toward their expected values.

The TRACC‐PHYSIO method contains certain limitations. First, it relies on high‐quality peripheral physiological recordings. Suboptimal sensor placement or subject motion can degrade physiological signals and reduce Peak CorrCoeff values. This can be addressed with rigorous quality control of physiological data during scanning, as well as postprocessing steps such as denoising and outlier detection. A final visual quality check is recommended to exclude physiological recordings that cannot be adequately corrected. Second, the Peak CorrCoeff depends on the similarity between the physiological and MR signals. Variations in MR waveform shape may arise from system‐specific transfer functions that describe how physiological inputs are transformed into the measured MRI signal. Deviations from an ideal transfer (i.e., a perfect impulse response) reduce the maximum achievable Peak CorrCoeff. Because these transfer functions are not well characterized in neurofluid compartments, improved characterization could refine the TRACC‐PHYSIO framework by providing insight into the expected maximal Peak CorrCoeff. Third, the TRACC‐PHYSIO method involves aligning MR slice timing with the reference physiological signal; this alignment restricts the application of motion correction, as it would disrupt slice timings and this alignment. In the future, it may be possible to apply motion correction to both the MR signal and the physiological slice timing vectors, which will increase the Peak CorrCoeff and provide more accurate estimates for the TimeDelay; however, this approach may introduce interpolation errors. These limitations introduce both nonphysiological and physiological factors that may reduce Peak CorrCoeff, thereby reducing the certainty of TimeDelay estimation.

In conclusion, our simulations support that TRACC‐PHYSIO is a practical and reliable time‐domain approach to quantify cardiac and respiratory coupling, as well as their pulse delays with high millisecond‐scale temporal resolution. This method is particularly valuable in long‐TR MRI acquisitions, where frequency‐domain analyses are not feasible and physiological oscillations are obscured by cardiac aliasing. Its application to both existing and future datasets may provide important insights into the mechanisms of brain pulsations and their alterations in health and disease.

## Funding

Multiple National Institutes of Health grants supported this work, including funding from the National Institute on Aging: F30AG084336 (PI: Adam Wright) and RF1AG083762 (PI: Qiuting Wen). Support was provided by the National Institute of General Medical Sciences under Award Number T32GM148382.

## Supporting information


**Video S1:** Overview of the TRACC‐PHYSIO method for measuring peak cardiac coupling and pulse time delay. The left panel illustrates the MR and PPG signals at different Time Shifts. The right panel shows the corresponding CorrCoeff at each Time Shift, illustrating how the TRACC‐Cardiac waveform is generated and used to derive the Peak CorrCoeff and TimeDelay. The MR signal is not interpolated.

**Figure S1:** Linear relationship between TRACC‐PHYSIO Peak CorrCoeff and relative physiological bandpower in cerebral gray matter (GM) using fast fMRI. (A) Representative single‐voxel GM fMRI time series (top), its bandpower spectrum (left), and TRACC‐PHYSIO waveforms (right). (B) The mean Peak CorrCoeff versus relative bandpower for cardiac (red) and respiratory (blue) components in all participants. Card & C, cardiac; Resp & R, respiratory. **p < 0.01, ***p < 0.001.
**Figure S2:** TRACC‐PHYSIO demonstrated robust performance across varying TRs and acquisition times when the target had equal physiological component in the MR signal. Top row (A–D): TRACC‐Cardiac results for MR signals with C:*R* = 1:1. Bottom row (E–H): TRACC‐Respiratory results for MR signals with C:*R* = 1:1. From left to right: (A and E) Example synthetic MR signals with TR = 50 ms. (B and F) The mean (solid line) and standard deviation (shaded) of the Peak CorrCoeff and TimeDelay error with increasing TRs and a fixed acquisition time of 300 s. (C and G) The mean (solid line) and standard deviation (shaded) of the Peak CorrCoeff and TimeDelay error with increasing acquisition time and a fixed TR of 2 s. (D and H) Heatmap of the mean absolute TimeDelay error across all combinations of TRs and acquisition times. 5000 permutations were completed for each combination of TR and acquisition time. C:R, cardiac‐to‐respiratory ratios.
**Figure S3:** Heatmaps of mean absolute Peak CorrCoeff error in TRACC‐Cardiac with varying MR repetition times (TR), acquisition times, and MR signal physiological components. The mean absolute Peak CorrCoeff remained stable across large ranges of TRs and acquisition times in MR signals with different physiological components (A) C:*R* = 2:1, (B) C:*R* = 1:1, and (C) C:R = 1:2.
**Figure S4:** Heatmap of the mean absolute Peak CorrCoeff error in TRACC‐Respiratory with varying MR repetition times (TR), acquisition times, and MR signal physiological components. The mean absolute Peak CorrCoeff error remained stable across large ranges of TRs and acquisition times in MR signals with different physiological components (A) C:*R* = 1:2, (B) C:*R* = 1:1, and (C) C:R = 2:1.
**Figure S5:** The influence of the MR signal cardiac: respiratory ratio (C:R) and repetition time (TR) in TRACC‐Cardiac measurements, with a fixed acquisition time of 300 s. The Peak CorrCoeff and TimeDelay error across varying C:R at, (A) TR = 50 ms and (B) TR = 2 s. (C) The mean Peak CorrCoeff and (D) mean TimeDelay error across each combination of C:R and TR.
**Figure S6:** The influence of the MR signal cardiac: respiratory ratio (C:R) and repetition time (TR) in TRACC‐Respiratory measurements, with a fixed acquisition time of 300 s. The Peak CorrCoeff and TimeDelay error across varying C:R at, (A) TR = 50 ms and (B) TR = 2 s. (C) The mean Peak CorrCoeff and (D) mean TimeDelay error across each combination of C:R and TR.
**Table S1:** The relationship of absolute TimeDelay error with the absolute Peak CorrCoeff and repetition time (TR) determined with a linear mixed effects model using every simulated combination of physiological ratio and TR with TRACC‐Cardiac.
**Table S2:** The relationship of absolute TimeDelay error with the absolute Peak CorrCoeff and repetition time (TR) determined with a linear mixed effects model using every simulated combination of physiological ratio and TR with TRACC‐Respiratory.

## Data Availability

Code for the TRACC‐PHYSIO method and the simulated signal generation are available on GitHub (https://github.com/Wen‐Imaging‐Lab/TRACC_PHYSIO). Code for implementation with real data is also provided, along with an example TRACC‐Cardiac dataset available on Zenodo (10.5281/zenodo.18762411">https://doi.org/10.5281/zenodo.18762411">10.5281/zenodo.18762411).

## Appendix A

TRACC‐PHYSIO can be expressed mathematically as follows. The CorrCoeff is computed across a range of temporal lags (τ) as (Equation A1):

(A1)
$$\text{CorrCoeff}\left(\tau \right)=\frac{1}{N-1}\sum_{n=1}^{N}{\mathrm{MR}}_{Z}\left(n\right)\cdot {\mathrm{PPG}}_{Z}\left(t_{n}+\tau \right)$$

where the MR_Z_ and PPG_Z_ denote z‐scored MR and lagged physiological signals, respectively, and N is the number of MR acquisition timepoints. The Peak CorrCoeff is defined as the maximum absolute CorrCoeff (Equation A2):

(A2)
Peak CorrCoeff=max(|CorrCoeff|)

The corresponding TimeDelay is defined as the lag value (τ) that maximizes the absolute CorrCoeff (Equation A3):

(A3)
$$\text{TimeDelay}=\underset{\tau }{\arg\;\max}\left|\text{CorrCoeff}\left(\tau \right)\right|$$
