# Supplementary material for: TRACC‐PHYSIO: Time‐Domain Resolution‐Aligned Cross‐Correlation to Estimate PHYSIOlogical Coupling and Time Delays in Dynamic MRI
Source: Magn Reson Med. 2026 May 24;96(4):1605–16. doi: 10.1002/mrm.70446 (PMC13419326; doi:10.1002/mrm.70446)
Supplement: Supplementary file 2 — Figure S1: Linear relationship between TRACC‐PHYSIO Peak CorrCoeff and relative physiological bandpower in cerebral gray matter (GM) using fast fMRI. (A) Representative single‐voxel GM fMRI time series (top), its bandpower spectrum (left), and TRACC‐PHYSIO waveforms (right). (B) The mean Peak CorrCoeff versus relative bandpower for cardiac (red) and respiratory (blue) components in all participants. Card & C, cardiac; Resp & R, respiratory. **p < 0.01, ***p < 0.001. Figure S2: TRACC‐PHYSIO demonstrated robust performance across varying TRs and acquisition times when the target had equal physiological component in the MR signal. Top row (A–D): TRACC‐Cardiac results for MR signals with C:R = 1:1. Bottom row (E–H): TRACC‐Respiratory results for MR signals with C:R = 1:1. From left to right: (A and E) Example synthetic MR signals with TR = 50 ms. (B and F) The mean (solid line) and standard deviation (shaded) of the Peak CorrCoeff and TimeDelay error with increasing TRs and a fixed acquisition time of 300 s. (C and G) The mean (solid line) and standard deviation (shaded) of the Peak CorrCoeff and TimeDelay error with increasing acquisition time and a fixed TR of 2 s. (D and H) Heatmap of the mean absolute TimeDelay error across all combinations of TRs and acquisition times. 5000 permutations were completed for each combination of TR and acquisition time. C:R, cardiac‐to‐respiratory ratios. Figure S3: Heatmaps of mean absolute Peak CorrCoeff error in TRACC‐Cardiac with varying MR repetition times (TR), acquisition times, and MR signal physiological components. The mean absolute Peak CorrCoeff remained stable across large ranges of TRs and acquisition times in MR signals with different physiological components (A) C:R = 2:1, (B) C:R = 1:1, and (C) C:R = 1:2. Figure S4: Heatmap of the mean absolute Peak CorrCoeff error in TRACC‐Respiratory with varying MR repetition times (TR), acquisition times, and MR signal physiological components. The mean absolute Peak CorrCoeff erro [file MRM-96-1605-s002.docx]

**Supplemental Material:**


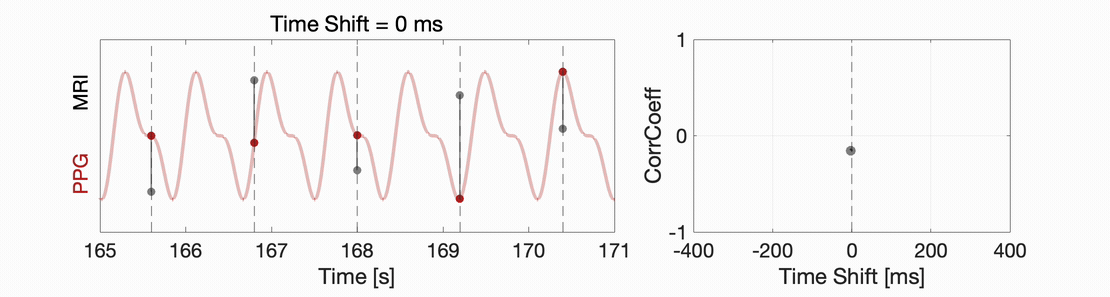


**Video S1: Overview of the TRACC-PHYSIO method for measuring peak cardiac coupling and pulse time delay.** The left panel illustrates the MR and PPG signals at different Time Shifts. The right panel shows the corresponding CorrCoeff at each Time Shift, illustrating how the TRACC-Cardiac waveform is generated and used to derive the Peak CorrCoeff and TimeDelay. **Note:** The MR signal is not interpolated.


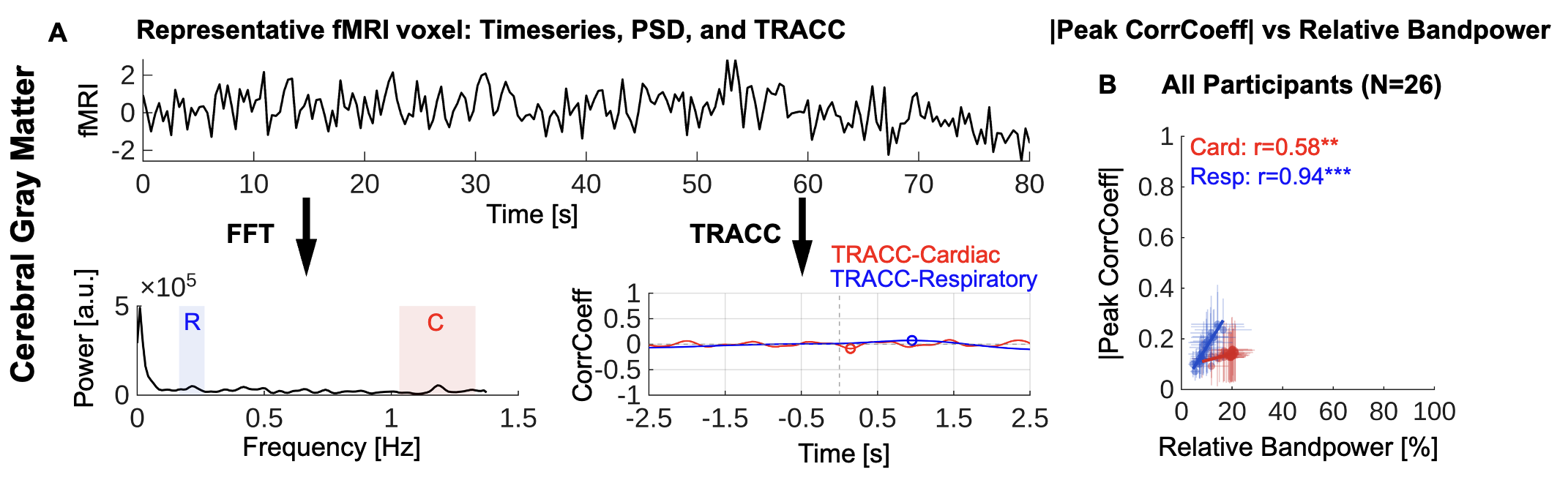


**Figure S1:** **Linear relationship between TRACC-PHYSIO Peak CorrCoeff and relative physiological bandpower in cerebral gray matter (GM) using fast fMRI.** (**A)** Representative single-voxel GM fMRI time series (top), its bandpower spectrum (left), and TRACC-PHYSIO waveforms (right). (**B**) The mean Peak CorrCoeff vs. relative bandpower for cardiac (red) and respiratory (blue) components in all participants. **Abbreviations:** Card & C – cardiac, Resp & R – respiratory. **Note:** **p<0.01, ***p<0.001.


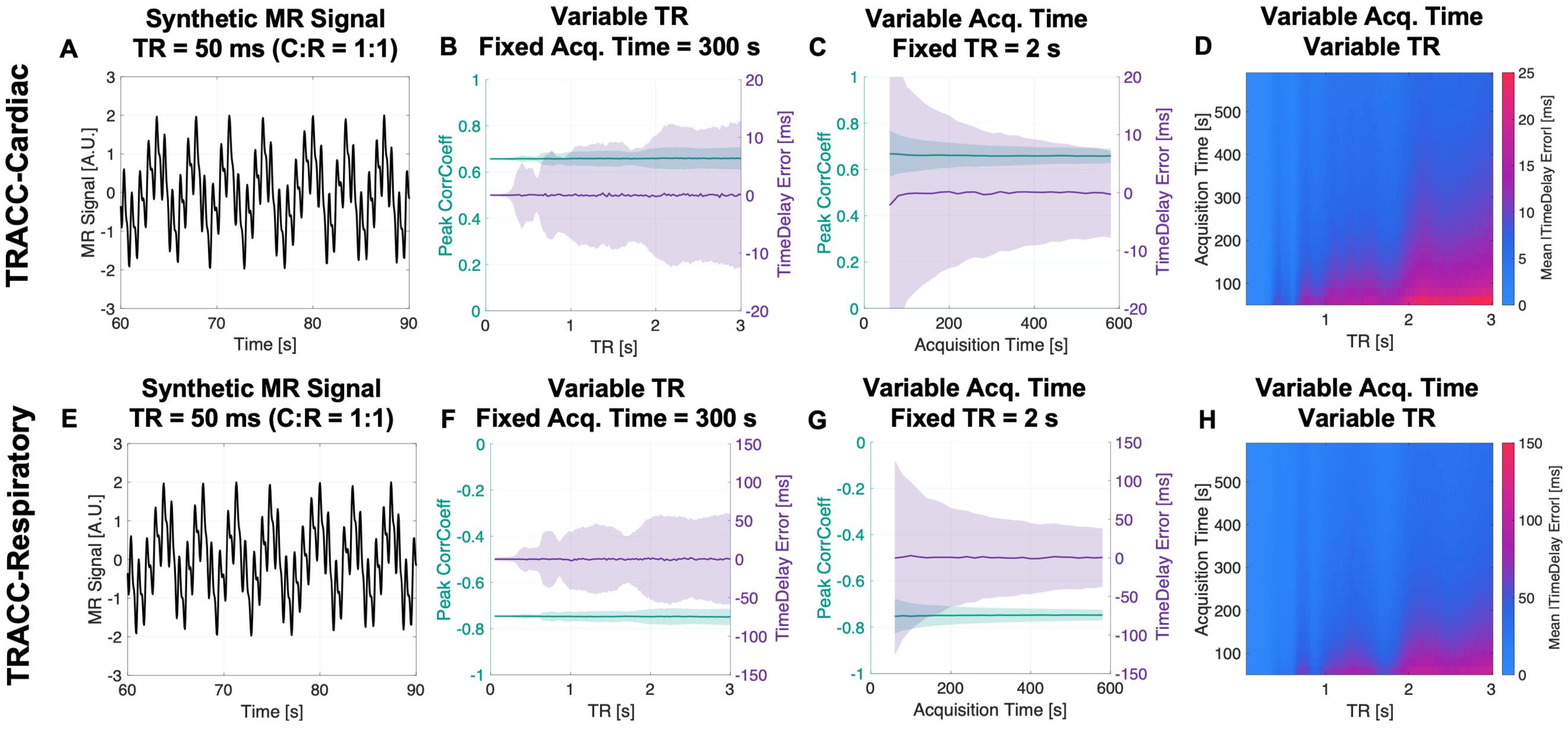


**Figure S2:** **TRACC-PHYSIO demonstrated robust performance across varying TRs and acquisition times when the target had equal physiological component in the MR signal.** Top row (**A-D**): TRACC-Cardiac results for MR signals with C:R=1:1. Bottom row (**E-H**): TRACC-Respiratory results for MR signals with C:R=1:1. From left to right: (A & E) Example synthetic MR signals with TR = 50 ms. (**B & F**) The mean (solid line) and standard deviation (shaded) of the Peak CorrCoeff and TimeDelay error with increasing TRs and a fixed acquisition time of 300 s. (**C & G**) The mean (solid line) and standard deviation (shaded) of the Peak CorrCoeff and TimeDelay error with increasing acquisition time and a fixed TR of 2 s. (**D & H**) Heatmap of the mean absolute TimeDelay error across all combinations of TRs and acquisition times. **Note:** 5000 permutations were completed for each combination of TR and acquisition time. **Abbreviations:** C:R – cardiac-to-respiratory ratios.

To quantify CorrCoeff error, the ground truth was defined as the mean Peak CorrCoeff at the shortest TR (0.05 s) and longest acquisition time (580 s). The mean Peak CorrCoeff error was computed as the difference between the ground-truth and TRACC-estimated Peak CorrCoeff. The mean absolute Peak CorrCoeff error was minimal across all cardiac-respiratory ratios (C:R), TRs, and acquisition times in both TRACC-Cardiac (Figure S3) and TRACC-Respiratory (Figure S4).


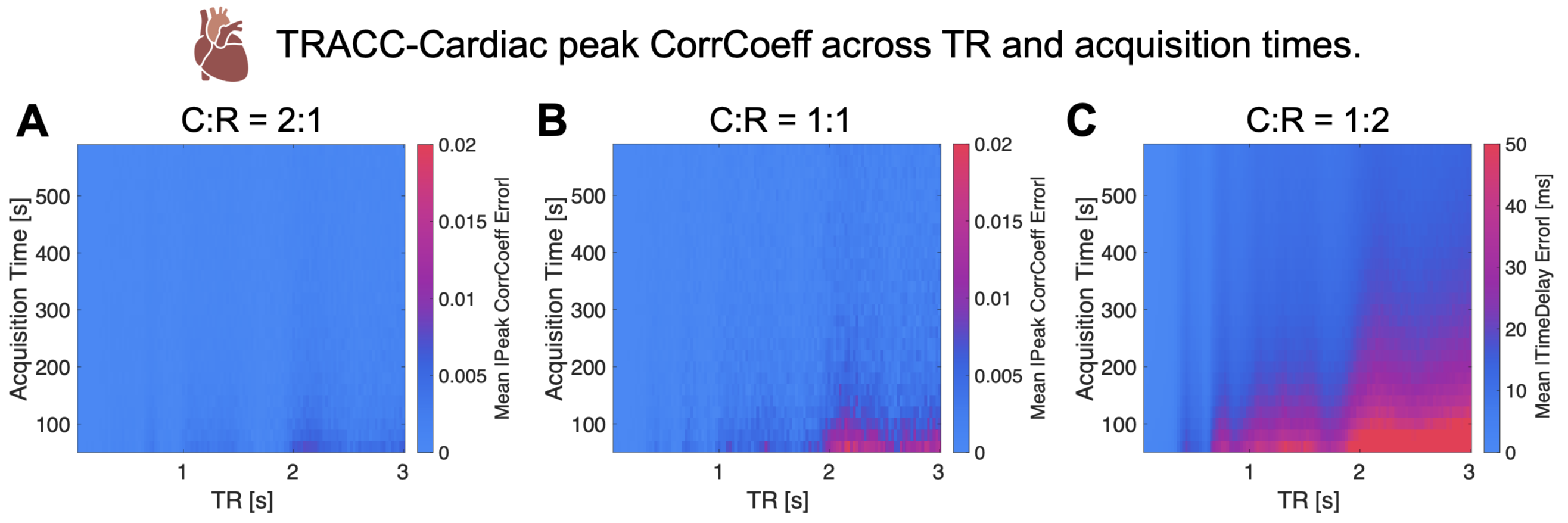


**Figure S3:** Heatmaps of mean absolute Peak CorrCoeff error in TRACC-Cardiac with varying MR repetition times (TR), acquisition times, and MR signal physiological components. The mean absolute Peak CorrCoeff remained stable across large ranges of TRs and acquisition times in MR signals with different physiological components (**A**) C:R = 2:1, (**B**) C:R = 1:1, and (**C**) C:R = 1:2.


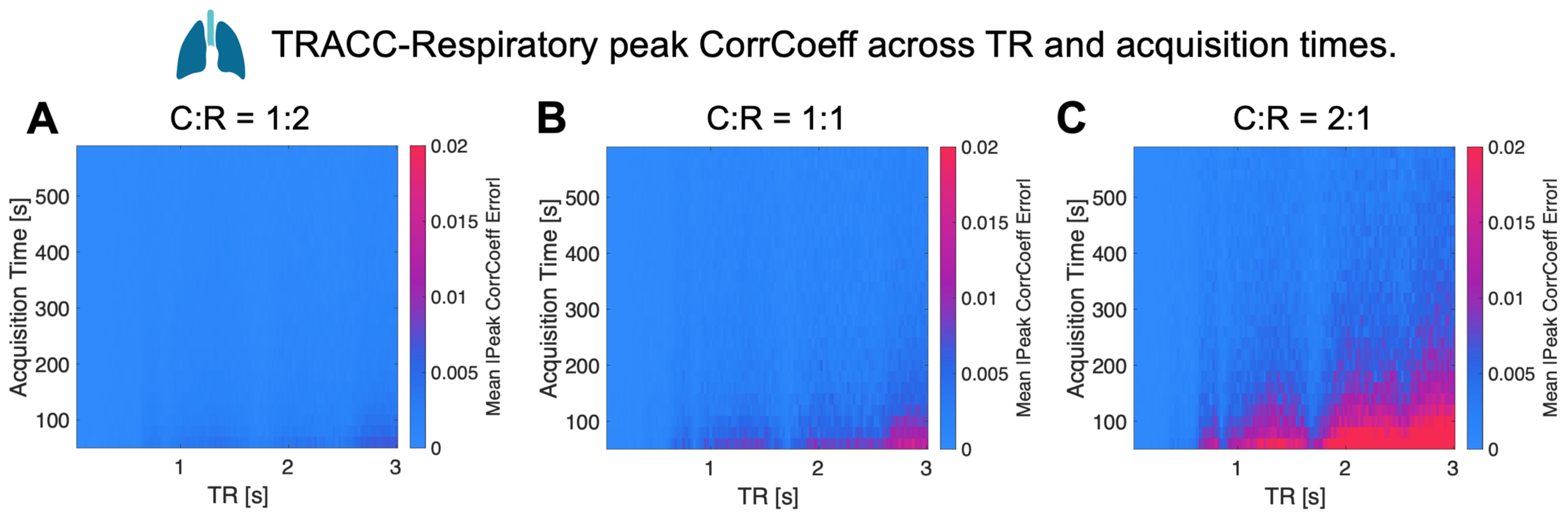


**Figure S4:** Heatmap of the mean absolute Peak CorrCoeff error in TRACC-Respiratory with varying MR repetition times (TR), acquisition times, and MR signal physiological components. The mean absolute Peak CorrCoeff error remained stable across large ranges of TRs and acquisition times in MR signals with different physiological components (**A**) C:R = 1:2, (**B**) C:R = 1:1, and (**C**) C:R = 2:1.

**In-depth simulations of TRACC-PHYSIO with varying cardiac-to-respiratory ratios (C:R)**

To assess how the cardiac-to-respiratory ratio (C:R) influence Peak CorrCoeff and TimeDelay estimates, we performed simulations with varying C:R from 4:1 to 1:4 (in increments of 0.1; i.e. 4:1, 3.9:1, …, 1:1, 1:1.1, 1:1.2, …, 1:4) with a fixed acquisition time of 300 s and varying TR from 0.05 to 3 s (in increments of 0.05 s). For each combination of C:R and TR, 5,000 permutations were completed.

**TRACC-Cardiac Results:**

Using TRACC-Cardiac, the Peak CorrCoeff increased as the C:R increased (Figure S5). With a fast TR of 50 ms, no TimeDelay errors were observed across all C:R (Figure S5A). With a TR of 2 s, TimeDelay errors increased as the C:R decreased. At C:R < 1:3, the mean TimeDelay error deviated from 0, indicating a bias towards underestimation (Figure S5B). Across all TRs, the estimation of the Peak CorrCoeff was consistent for all C:R (Figure S5C). As expected, TimeDelay errors increased as the cardiac component became less dominant (lower C:R ratio) and with longer TR.


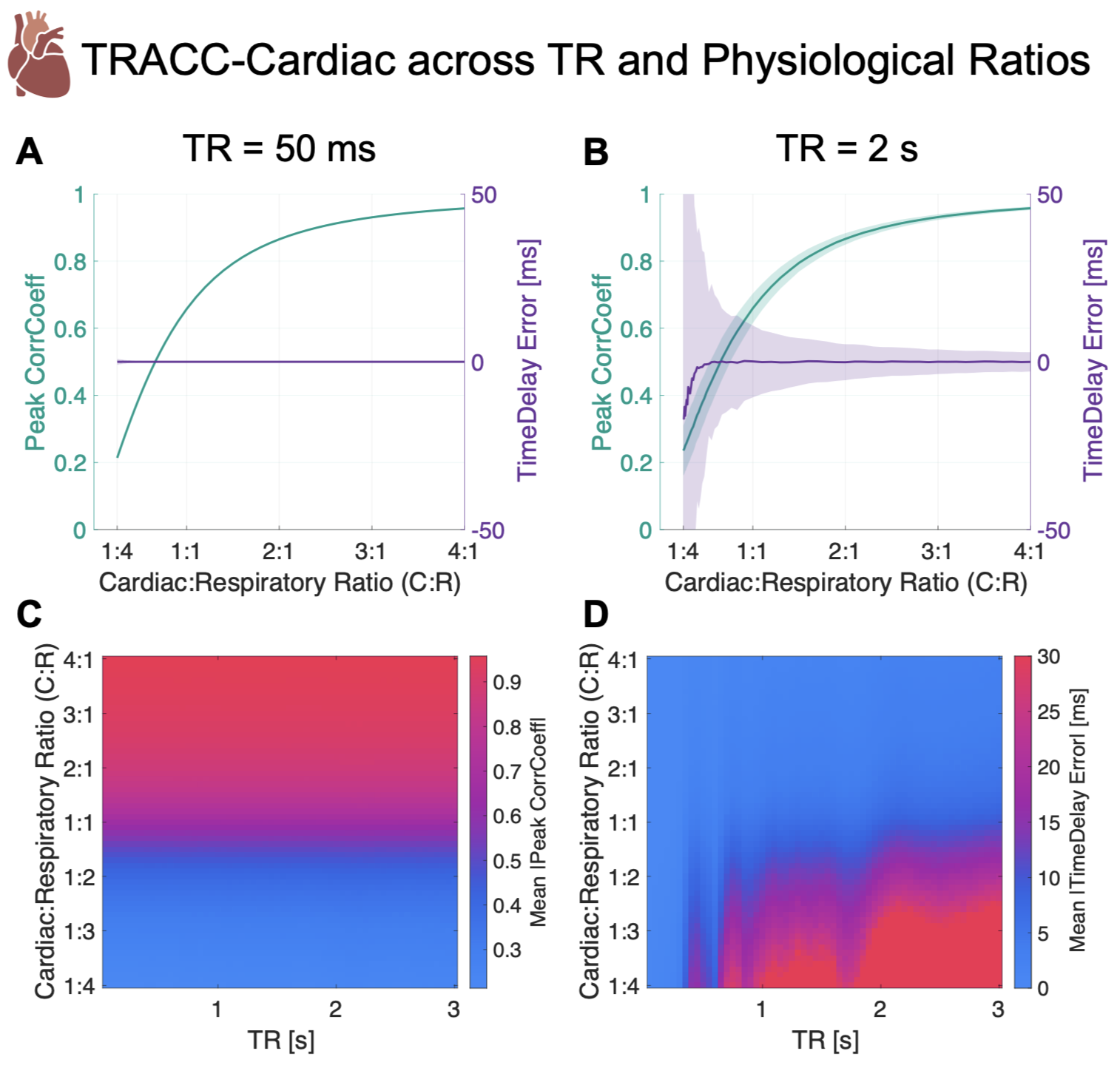


**Figure S5:** The influence of the MR signal cardiac:respiratory ratio (C:R) and repetition time (TR) in TRACC-Cardiac measurements, with a fixed acquisition time of 300 s. The Peak CorrCoeff and TimeDelay error across varying C:R at, (**A**) TR = 50 ms and (**B**) TR = 2 s. (**C**) The mean Peak CorrCoeff and (**D**) mean TimeDelay error across each combination of C:R and TR.

Variation in MR signal physiological components introduced changes in the Peak CorrCoeff, which initially increased nonlinearly and then gradually plateaued as the C:R ratio increased. Since the Peak CorrCoeff reflects the dominance of the physiological signal—and this dominance influences TimeDelay accuracy—CorrCoeff may serve as an indicator of confidence in the TimeDelay estimate. To evaluate the TimeDelay error vs Peak CorrCoeff relationship, we fit a linear mixed-effects model to assess the relationship between the absolute TimeDelay error, the absolute Peak CorrCoeff, and TR. Each of the 5000 permutations for each (TR, Physiological Ratio) pair were treated as a repeated measure (Repeat), using Equation 1.

|TimeDelay Error| ~ 1 + |Peak CorrCoeff| x TR + (1 | Repeat) (1)

The regression results showed that absolute TimeDelay error was negatively associated with absolute Peak CorrCoeff and positively associated with TR (Supplemental Table 1). A significant negative interaction between Peak CorrCoeff and TR indicated that the reduction in TimeDelay error observed with high Peak CorrCoeff was stronger at longer TRs. In other words, at longer TRs, the Peak CorrCoeff was a stronger predictor of TimeDelay error.

**Table S1:** The relationship of absolute TimeDelay error with the absolute Peak CorrCoeff and repetition time (TR) determined with a linear mixed effects model using every simulated combination of physiological ratio and TR with TRACC-Cardiac.

| **Term** | **Estimate (95% CI)** | **SE** | **t-stat** | **p-value** |
| --- | --- | --- | --- | --- |
| (Intercept) | 23.8 (22.6, 25.0) | 0.62 | 38.7 | p<0.001 |
| \|Peak CorrCoeff\| | -35.6 (-36.5, -34.7) | 0.46 | -35.6 | p<0.001 |
| TR | 27.6 (27.0, 28.3) | 0.34 | 81.7 | p<0.001 |
| \|Peak CorrCoeff\| × TR | -34.7 (-35.1, -34.3) | 0.22 | -161.3 | p<0.001 |

**TRACC-Respiratory Results:**

Using TRACC-Respiratory, the Peak CorrCoeff increased as C:R decreased (i.e., the respiratory component increased, Figure S6). With a fast TR of 50 ms, no TimeDelay errors were experienced across all C:R (Figure S6A). With a TR of 2 s, variations in TimeDelay errors increased as C:R increased, and no significant error bias was observed at any C:R (Figure S6B). Across all TRs, the estimation of the Peak CorrCoeff was consistent for all C:R (Figure S6C). Minimal TimeDelay errors were observed with TRs<600 ms for all C:R (Figure S5D). For TRs>600 ms, minimal TimeDelay errors were observed in signals with equal physiological components (C:R = 1) or signals dominated by respiratory amplitude (C:R<1, Figure S6D). Increased TimeDelay errors were present when TRs exceeded 1 s and C:R>2 (Figure S6D). As expected, TimeDelay errors increased as the respiratory component was less dominant (higher C:R ratio) and with longer TR.


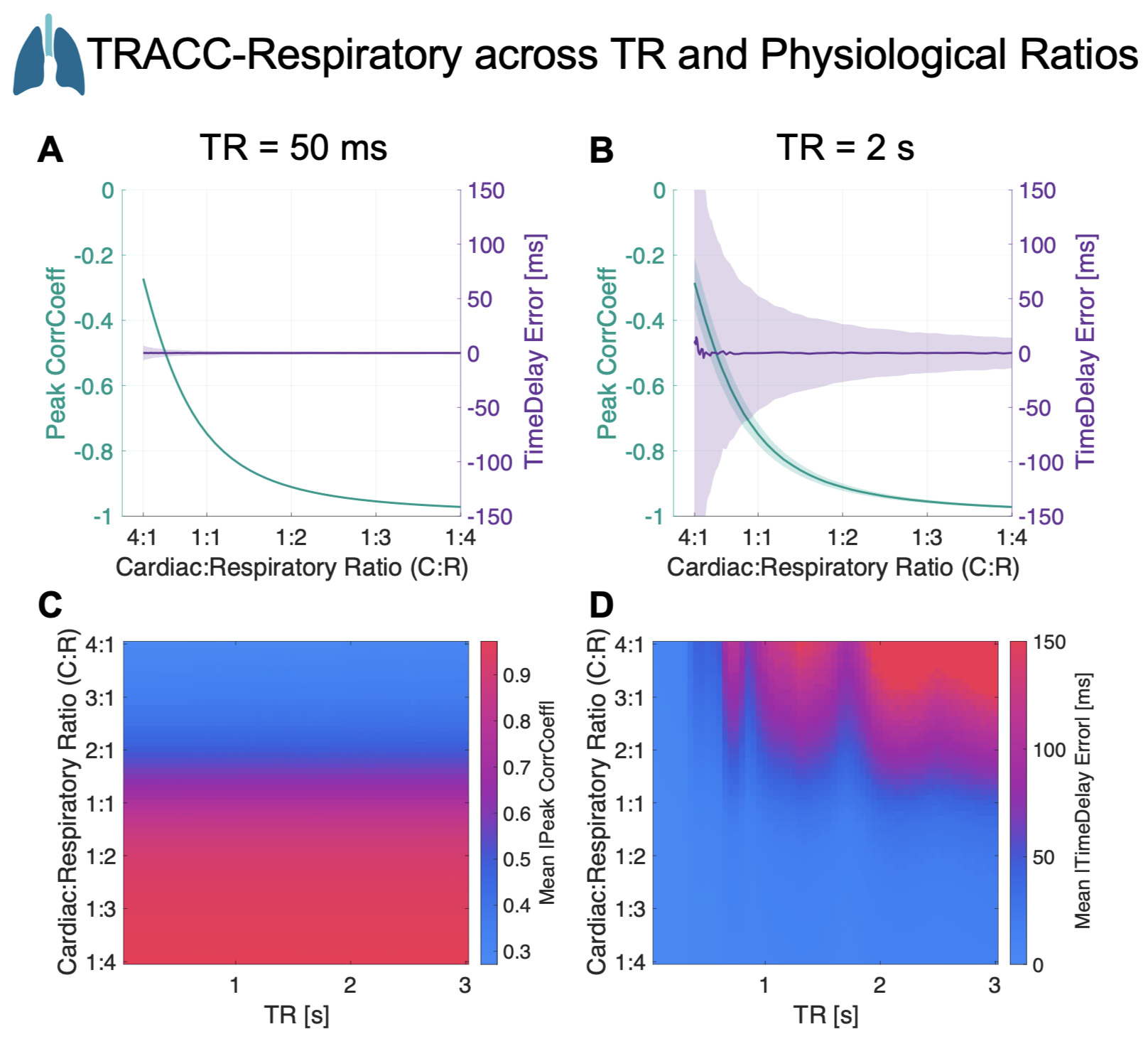


**Figure S6:** The influence of the MR signal cardiac:respiratory ratio (C:R) and repetition time (TR) in TRACC-Respiratory measurements, with a fixed acquisition time of 300 s. The Peak CorrCoeff and TimeDelay error across varying C:R at, (**A**) TR = 50 ms and (**B**) TR = 2 s. (**C**) The mean Peak CorrCoeff and (**D**) mean TimeDelay error across each combination of C:R and TR.

The modeling of TimeDelay errors in TRACC-Respiratory behaved similarly to TRACC-Cardiac. The absolute TimeDelay error was negatively associated with absolute Peak CorrCoeff and positively associated with TR. Additionally, a negative interaction between Peak CorrCoeff and TR indicated that the reduction in TimeDelay error with higher Peak CorrCoeff was more pronounced at longer TRs (Supplemental Table 2). In other words, at longer TRs, the Peak CorrCoeff was a stronger predictor of TimeDelay error.

**Table S2:** The relationship of absolute TimeDelay error with the absolute Peak CorrCoeff and repetition time (TR) determined with a linear mixed effects model using every simulated combination of physiological ratio and TR with TRACC-Respiratory.

| **Term** | | **Estimate (95% CI)** | **SE** | **t-stat** | **p-value** |
| --- | --- | --- | --- | --- | --- |
| (Intercept) | 44.7 (42.7, 46.7) | | 1.02 | 43.7 | p<0.001 |
| \|Peak CorrCoeff\| | -51.6 (-53.9, -49.2) | | 1.19 | -43.3 | p<0.001 |
| TR | 81.8 (80.8, 82.9) | | 0.52 | 156.0 | p<0.001 |
| \|Peak CorrCoeff\| × TR | -86.5 (-87.6, -85.4) | | 0.57 | -152.9 | p<0.001 |
